# Exploring the prognostic value of the novel nutritional index for in-hospital mortality in acute coronary syndrome: a sex-specific analysis

**DOI:** 10.3389/fmed.2025.1498260

**Published:** 2025-04-24

**Authors:** Guimei Li, Shujuan Li

**Affiliations:** ^1^Geriatric Center, Inner Mongolia Medical University Affiliated Hospital, Hohhot, Inner Mongolia, China; ^2^Department of Cardiovascular Medicine, Inner Mongolia Medical University Affiliated Hospital, Hohhot, Inner Mongolia, China

**Keywords:** acute coronary syndrome, nutritional index, TCBI, in-hospital mortality, prognosis

## Abstract

**Background:**

Emerging evidence suggests that nutritional status plays a pivotal role in determining the prognosis of patients with acute coronary syndrome (ACS). This study aimed to investigate the relationship between a novel nutritional index, Triglycerides × Total Cholesterol × Body Weight Index (TCBI), and short-term prognosis in patients with ACS.

**Methods:**

A retrospective study was conducted using data from 5,277 ACS patients admitted to intensive care units of 208 United States hospitals in the eICU Collaborative Research Database (eICU-CRD) in 2014 and 2015. Patients were divided into three groups based on TCBI tertiles: Group 1 (< 1017.97), Group 2 (1017.97–2069.02), and Group 3 (> 2069.02).

**Results:**

Multivariate logistic regression analysis showed that after adjusting for 17 confounding variables, higher TCBI had significantly lower in-hospital mortality [Tertile 3 vs Tertile 1: OR (95% CI): 0.67 (0.48, 0.94), *p* = 0.019]. This relationship was significant in the male subgroup but not in the female subgroup. The association between TCBI and in-hospital mortality was more pronounced in male patients and those with blood pressure > 140 mmHg. Subgroup analysis revealed a significant interaction between sex and the predictive value of TCBI (*p* for interaction < 0.05).

**Conclusion:**

Higher TCBI was independently associated with decreased in-hospital mortality in ACS patients, particularly in male patients. TCBI, as a novel nutritional index, may serve as a practical tool for risk stratification and personalized management of ACS patients.

## Introduction

Acute coronary syndrome (ACS), which includes acute myocardial infarction and unstable angina, poses a significant global health burden. The incidence and mortality rates of ACS vary across countries, with higher rates observed in developing nations due to the adoption of Western diets and sedentary lifestyles (1). ACS not only leads to substantial morbidity and mortality but also imposes a heavy economic burden on healthcare systems worldwide. Patients with ACS have a poor prognosis, with high rates of recurrent cardiovascular events, heart failure, and death (2).

Traditional prognostic indicators for ACS, such as the Global Registry of Acute Coronary Events (GRACE) score and the Thrombolysis in Myocardial Infarction (TIMI) risk score, are widely used to predict outcomes. However, these scores have limitations, as they do not fully capture patients’ nutritional status, which significantly impacts prognosis. Malnutrition is common in ACS patients and is strongly associated with increased mortality and cardiovascular events (3). The Geriatric Nutritional Risk Index (GNRI) and Prognostic Nutritional Index (PNI) have shown promise in predicting outcomes in coronary heart disease patients (4, 5). However, due to the complexity of nutritional assessment, there is no universally accepted nutritional index for clinical use in cardiovascular patients.

Triglycerides × Total Cholesterol × Body Weight Index (TCBI) is a novel nutritional index that combines triglycerides, total cholesterol, and body weight (6). TCBI integrates three key parameters that collectively reflect nutritional status. Physiologically, triglycerides represent the primary form of stored energy and can indicate adequate caloric reserves (7). Total cholesterol serves as an essential component for cell membrane integrity, hormone synthesis, and neurological function (8). Body weight provides a comprehensive indicator of overall nutritional balance and energy stores. During acute cardiovascular events like ACS, these nutritional parameters become particularly relevant as patients experience heightened metabolic stress and catabolism. The “obesity paradox” observed in cardiovascular patients suggests that modest elevations in body weight and certain lipid parameters may confer short-term survival advantages (9), potentially by providing crucial energy substrates when the myocardium is under ischemic stress and requires alternative metabolic resources. By combining these three parameters, TCBI offers a more comprehensive assessment of nutritional status than any single component alone, potentially capturing both energy reserves and metabolic capacity that could influence clinical outcomes in ACS patients.

Based on the physiological functions of its components, the prognostic value of TCBI in cardiovascular disease has been preliminarily explored in prior studies. For example, Shinichiro Doi et al. found that elevated TCBI was associated with lower rates of all-cause mortality, cardiovascular mortality, and cancer mortality in patients undergoing percutaneous coronary intervention (PCI) (6). Furthermore, Maruyama et al. discovered that low TCBI significantly predicted the incidence of major adverse cardiac and cerebrovascular events in elderly patients after PCI (10). Given the differences in nutrition and metabolism between men and women, such as higher body fat percentage and lower muscle mass in women (11), the predictive value of TCBI may vary between sexes. However, no studies have explored this aspect, which is crucial for the ACS population.

Therefore, we conducted this study to investigate the relationship between TCBI and short-term prognosis in ACS patients, stratified by sex. By examining this association in a large, diverse ACS population using data from the eICU Collaborative Research Database (eICU-CRD), we aimed to investigate the prognostic value of TCBI in an ACS population, with a particular focus on exploring the differences in its prognostic impact across different sexes. The findings of this study may have important implications for the risk stratification and personalized management of ACS patients, ultimately improving their outcomes and reducing the societal burden of this devastating condition.

## Materials and methods

### Participants and study design

This study utilized the eICU-CRD, a multi-center intensive care unit (ICU) database comprising over 200,000 admission cases from 208 hospitals and 335 ICUs across the United States in 2014 and 2015 (12). The database encompassed comprehensive, high-resolution clinical data, including vital signs, laboratory measurements, medication records, Acute Physiology and Chronic Health Evaluation components, care plan details, admission diagnoses, and patient medical histories. Additionally, it contained time-stamped diagnoses from structured problem lists and corresponding treatment selections. This database was released in compliance with the Health Insurance Portability and Accountability Act (HIPAA) safe harbor provision. The re-identification risk was independently assessed and certified as meeting safe harbor standards by Privacert (Cambridge, MA) (HIPAA Certification no. 1031219-2). To gain access to this valuable resource, our research team completed the Protecting Human Research Participants examination, ensuring adherence to ethical standards in human subject research (Certificate number: 9728458).

**FIGURE 1:**
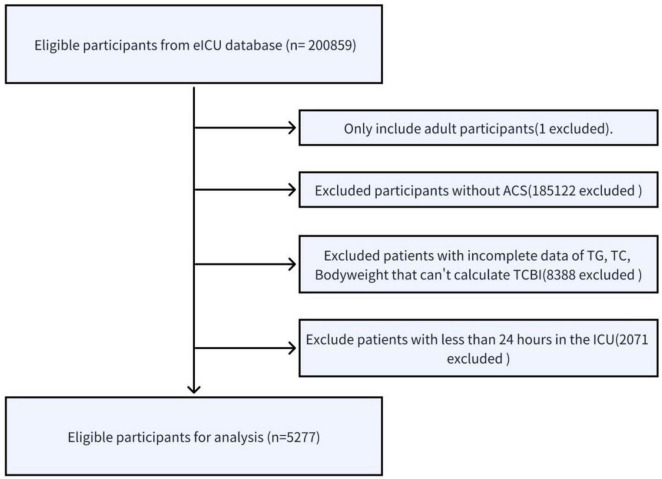
Flow diagram of the patient selection process. ACS, acute coronary syndrome; TG, triglycerides; TC, total cholesterol; TCBI, Triglycerides × Total Cholesterol × Body Weight Index; ICU, intensive care unit.

Patients diagnosed with acute coronary syndrome (ACS) based on International Classification of Diseases, Ninth Revision (ICD-9) codes and admitted to the ICU for their first hospitalization were enrolled in this study. ACS was identified using ICD-9 codes 410.xx [acute myocardial infarction, including ST-elevation myocardial infarction (STEMI) and non-ST-elevation myocardial infarction (NSTEMI)] and 411.1 (unstable angina). Exclusion criteria were applied as follows: age below 18 years, non-ACS participants, incomplete data on triglycerides (TG), total cholesterol (TC), or body weight preventing calculation of the TCBI, and ICU length of stay less than 24 h. After screening, a total of 5,277 patients were included in the final analysis. The detailed patient selection and screening process is illustrated in Figure 1.

### Data collection

The following patient information was collected within 24 h of ICU admission: Demographic data included sex, age, and race. Anthropometric measurements comprised body mass index (BMI). Vital signs encompassed systolic blood pressure (SBP), diastolic blood pressure (DBP), heart rate, respiratory rate, and oxygen saturation (SaO2). Laboratory parameters consisted of complete blood count (red blood cells, platelets, hematocrit, hemoglobin, mean corpuscular volume (MCV), mean corpuscular hemoglobin (MCH), electrolytes (sodium, potassium, anion gap), renal function markers [blood urea nitrogen (BUN), creatinine (Cr)], metabolic indicators (glucose, albumin), liver enzymes [alanine aminotransferase (ALT), aspartate aminotransferase (AST)], and lipid profile [TG, TC, high-density lipoprotein (HDL)]. Comorbidities were documented, including cardiovascular (atrial fibrillation (AF), hypertension, cardiomyopathy, congestive heart failure (CHF), hypercholesterolemia), pulmonary [chronic obstructive pulmonary disease (COPD), pneumonia, pleural effusion, acute respiratory distress syndrome (ARDS), asthma], renal [chronic kidney disease (CKD), acute kidney injury (AKI)], neurological (stroke), and systemic conditions [diabetes mellitus (DM), sepsis, deep vein thrombosis]. Interventions were recorded, such as coronary stenting, coronary artery bypass grafting (CABG), percutaneous transluminal coronary angioplasty (PTCA), dialysis, and mechanical ventilation. Medication data included antiplatelet agents, warfarin, angiotensin-converting enzyme inhibitors/angiotensin receptor blockers (ACEI/ARB), statins, vasoactive drugs, vasopressin, albumin treatment, antibiotics, and bronchodilators. In-hospital death was also documented. TCBI was calculated using the formula: TCBI = TG (mg/dL) × TC (mg/dL) × Body Weight (kg)/1,000 (6). This normalization by 1,000 was applied to scale the resulting values to a more manageable range without affecting the relative relationships between measurements.

### Grouping and outcome

According to TCBI tertiles, all patients were divided into three groups: Low (median = 684.00, IQR: 497.36–853.88, *n* = 1,759), Middle (median = 1,419.40, IQR: 1215.23–1722.52, *n* = 1,759), High (median = 3240.91, IQR: 2518.47–4591.06, *n* = 1,759). The study’s primary outcome was in-hospital death, which was defined as death occurring during hospitalization.

### Statistical analysis

Continuous variables were expressed as mean ± standard deviation (SD) or median (T1–T3) based on the distribution of data. In the test for differences, data with a normal distribution were analyzed using ANOVA analysis; for data with a skewed distribution, the Kruskal-Wallis H test was used. All categorical data were expressed as frequency (percentile). Chi-square test or Fisher test were applied to compare the categorical variables.

Logistic regression analysis was employed to explore the multivariate associations between TCBI and in-hospital death, first within the overall population and then stratified by sex. The results were expressed as odds ratios (OR) with 95% confidence intervals (CI). Model 1 was unadjusted, while Model 2 accounted for sex, age, and race. Model 3 was further adjusted for additional parameters, including heart rate, respiratory rate, CHF, COPD, CKD, DM, stroke, hemoglobin, Cr, BUN, sodium, antiplatelet agents, ACEI /ARBs, and statins. The covariates included in Model 3 were selected through a two-step process. First, we identified clinically relevant variables known to influence ACS outcomes based on previous literature and clinical expertise. Second, we applied stepwise regression to this clinically informed variable set, retaining only those variables showing a significance level of *p* < 0.05. Finally, to ensure model stability, we performed variance inflation factor (VIF) analysis, including only variables with VIF values less than five as covariates in the final model to minimize multicollinearity. To evaluate the goodness-of-fit of our logistic regression models, we employed the Hosmer-Lemeshow test to assess calibration and calculated Nagelkerke R^2^ values to measure the model’s explanatory power.

In the overall population and in different sex groups, the LOWESS (locally weighted scatter plot smoothing) curves were separately used to visually represent the correlation between TCBI levels as a continuous variable and in-hospital death. Subgroup analysis was conducted to estimate the consistency of the effect in different groups including age (≤ 65, > 65 years), sex, race, DM and SBP (≤ 140, > 140 mmHg), with results visualized using forest plots.

Statistical significance was defined as a two-tailed *p*-value of less than 0.05. All statistical analyses were performed using the R software environment (Version 4.3.2; The R Foundation^1^).

## Results

### Baseline characteristics of the participants

A total of 200,859 participants from the eICU database were initially enrolled in the study, and among these, 5,277 individuals met the eligibility criteria and were included in the analysis (Figure 1). There were 3,393 (64.30%) males and 1,884 (35.70%) females, and the average age of the participants was 65.24 ± 13.26. Ultimately, 341 (6.46%) participants experienced in-hospital death.

The baseline characteristics were shown in Table 1. Participants in the higher TCBI group were more likely to be male and have higher levels of BMI, SBP, DBP, platelets, sodium, glucose, hematocrit, hemoglobin, red blood cell, albumin, TG, TC, PTCA, and ACEI/ARB, but lower levels of heart rate, respiration, BUN, CR, Cr, MCV, ALT, HDL, AF, cardiomyopathy, CHF, COPD, Pneumonia, CKD, AKI, stroke, sepsis, deep vein thrombosis, dialysis, antibiotic and bronchodilator than those in the lower TCBI groups (all *p* < 0.001). Furthermore, in higher tertiles of TCBI, a notably lower rate of in-hospital death in ACS participants was observed (*p* < 0.001). There was no statistically significant difference in other indicators among different groups (all *p* > 0.05).

**TABLE 1:** Baseline characteristics of acute coronary syndrome (ACS) patients grouped by tertiles of Triglycerides × Total Cholesterol × Body Weight Index (TCBI).

Variables	Total	Low	Middle	High	*P*-value
*N*	5,277	1,759	1,759	1,759	–
TCBI, median (IQR)	1419.40 (853.90–2518.26)	684.00 (497.36–853.88)	1419.40 (1215.23–1722.52)	3240.91 (2518.47–4591.06)	< 0.001
Age (year), mean ± SD	65.24 ± 13.26	70.66 ± 12.73	65.18 ± 12.69	59.89 ± 12.10	< 0.001
Sex (*N*, %)	–	–	–	–	< 0.001
Male	3,393 (64.30%)	1,041 (59.18%)	1,118 (63.56%)	1,234 (70.15%)	–
Female	1,884 (35.70%)	718 (40.82%)	641 (36.44%)	525 (29.85%)	–
Race (*N*, %)	–	–	–	–	0.005
Caucasian	4,082 (77.35%)	1,338 (76.07%)	1,361 (77.37%)	1,383 (78.62%)	–
African American	500 (9.48%)	196 (11.14%)	173 (9.84%)	131 (7.45%)	–
Hispanic	695 (13.17%)	225 (12.79%)	225 (12.79%)	245 (13.93%)	–
BMI (kg/m^2^), mean ± SD	29.35 ± 6.91	26.04 ± 5.45	29.60 ± 6.56	32.40 ± 7.08	< 0.001
Vital sign	–	–	–	–	–
SBP (mmHg), mean ± SD	94.04 ± 29.14	90.15 ± 29.46	94.90 ± 29.12	97.05 ± 28.42	< 0.001
DBP (mmHg), mean ± SD	72.97 ± 17.91	70.01 ± 18.94	74.01 ± 17.94	74.88 ± 16.38	< 0.001
Heart rate, mean ± SD	81.97 ± 18.49	83.94 ± 19.90	81.20 ± 18.11	80.79 ± 17.20	< 0.001
Respiration, mean ± SD	19.69 ± 6.72	20.48 ± 6.92	19.67 ± 6.91	18.91 ± 6.24	< 0.001
Sao2, mean ± SD	96.76 ± 4.40	96.56 ± 4.91	96.95 ± 3.96	96.78 ± 4.28	0.032
**Laboratory**					
Red blood cell ( × 10ł/mL), mean ± SD	11.52 ± 6.28	11.45 ± 6.47	11.80 ± 7.41	11.30 ± 4.60	0.052
Platelets ( × 10ł/mL), median (IQR)	221.00 (179.00–269.00)	213.00 (169.00–266.00)	224.00 (184.00–272.50)	225.00 (186.00–268.00)	< 0.001
Sodium (mmol/L), mean ± SD	137.48 ± 5.48	137.11 ± 6.40	137.80 ± 4.76	137.53 ± 5.14	< 0.001
Potassium (mmol/L), mean ± SD	4.11 ± 0.63	4.14 ± 0.66	4.10 ± 0.63	4.08 ± 0.60	0.013
Anion gap (%), median (IQR)	11.55 ± 4.47	11.75 ± 4.74	11.32 ± 4.36	11.56 ± 4.28	0.040
BUN (mg/dL), mean ± SD	18.00 (13.85–25.00)	20.00 (15.00–29.00)	17.00 (13.23–25.00)	16.00 (13.00–22.00)	< 0.001
CR (mg/dL), median (IQR)	1.03 (0.83–1.35)	1.06 (0.83–1.46)	1.03 (0.82–1.33)	1.00 (0.83–1.26)	< 0.001
Glucose (mg/dL), median (IQR)	137.00 (112.00–190.00)	134.00 (109.00–174.00)	137.00 (112.00–189.00)	144.00 (114.00–210.00)	< 0.001
Creatinine (mg/dL), mean ± SD	1.35 ± 1.28	1.50 ± 1.48	1.31 ± 1.18	1.25 ± 1.14	< 0.001
Hematocrit (g/dL), mean ± SD	39.63 ± 6.44	37.30 ± 6.71	39.86 ± 6.15	41.72 ± 5.63	< 0.001
Hemoglobin (g/dL), mean ± SD	13.28 ± 2.34	12.39 ± 2.40	13.35 ± 2.24	14.10 ± 2.05	< 0.001
MCV (fl), mean ± SD	89.94 ± 6.03	90.60 ± 6.73	89.79 ± 5.93	89.43 ± 5.27	< 0.001
Red blood cell (m/mL), mean ± SD	4.42 ± 0.74	4.13 ± 0.76	4.45 ± 0.72	4.67 ± 0.65	< 0.001
MCH (pg), mean ± SD	30.07 ± 2.35	30.01 ± 2.62	30.02 ± 2.33	30.17 ± 2.06	0.103
Albumin (g/dl), mean ± SD	3.51 ± 0.59	3.33 ± 0.59	3.53 ± 0.58	3.67 ± 0.54	< 0.001
ALT (U/l), median (IQR)	29.00 (20.00–47.00)	28.00 (19.00–46.00)	29.00 (19.00–45.75)	32.00 (22.00–49.00)	< 0.001
AST (U/l), median (IQR)	36.00 (23.00–82.00)	37.00 (23.00–89.00)	37.00 (23.00–83.75)	35.00 (22.00–75.00)	0.076
TG (mg/dL), median (IQR)	113.00 (81.00–169.00)	72.00 (57.00–90.00)	111.00 (94.00–135.00)	198.00 (157.00–257.50)	< 0.001
TC (mg/dL), median (IQR)	152.00 (123.00–186.00)	123.00 (101.00–146.00)	153.00 (129.00–180.00)	185.00 (157.00–214.00)	< 0.001
HDL (mg/dL), median (IQR)	38.00 (31.00–47.00)	41.00 (32.00–52.00)	39.00 (32.00–47.00)	35.00 (29.00–42.00)	< 0.001
**Comorbidities, N (%)**					
AF	408 (7.73%)	193 (10.97%)	126 (7.16%)	89 (5.06%)	< 0.001
Hypertension	905 (17.15%)	294 (16.71%)	305 (17.34%)	306 (17.40%)	0.837
Cardiomyopathy	218 (4.13%)	102 (5.80%)	67 (3.81%)	49 (2.79%)	< 0.001
CHF	577 (10.93%)	242 (13.76%)	197 (11.20%)	138 (7.85%)	< 0.001
DM	709 (13.44%)	234 (13.30%)	219 (12.45%)	256 (14.55%)	0.184
Hypercholesterolemia	453 (8.58%)	142 (8.07%)	154 (8.75%)	157 (8.93%)	0.634
COPD	293 (5.55%)	130 (7.39%)	97 (5.51%)	66 (3.75%)	< 0.001
Pneumonia	328 (6.22%)	161 (9.15%)	94 (5.34%)	73 (4.15%)	< 0.001
Pleural effusion	63 (1.19%)	33 (1.88%)	19 (1.08%)	11 (0.63%)	0.003
ARDS	227 (4.30%)	99 (5.63%)	67 (3.81%)	61 (3.47%)	0.003
Asthma	71 (1.35%)	24 (1.36%)	21 (1.19%)	26 (1.48%)	0.762
CKD	415 (7.86%)	191 (10.86%)	124 (7.05%)	100 (5.69%)	< 0.001
AKI	520 (9.85%)	225 (12.79%)	163 (9.27%)	132 (7.50%)	< 0.001
Stroke	224 (4.24%)	101 (5.74%)	63 (3.58%)	60 (3.41%)	< 0.001
Sepsis	330 (6.25%)	148 (8.41%)	102 (5.80%)	80 (4.55%)	< 0.001
Deep vein thrombosis	32 (0.61%)	21 (1.19%)	4 (0.23%)	7 (0.40%)	< 0.001
**Interventions, N (%)**					
STENT	615 (11.65%)	165 (9.38%)	231 (13.13%)	219 (12.45%)	0.001
CABG	93 (1.76%)	26 (1.48%)	39 (2.22%)	28 (1.59%)	0.200
PTCA	899 (17.04%)	246 (13.99%)	332 (18.87%)	321 (18.25%)	< 0.001
Dialysis	153 (2.90%)	78 (4.43%)	44 (2.50%)	31 (1.76%)	< 0.001
Vent	857 (16.24%)	301 (17.11%)	278 (15.80%)	278 (15.80%)	0.479
**Medication, N (%)**					
Antiplatelet	3,344 (63.37%)	1,072 (60.94%)	1,134 (64.47%)	1,138 (64.70%)	0.035
Warfarin	264 (5.00%)	112 (6.37%)	75 (4.26%)	77 (4.38%)	0.006
ACEI/ARB	1,250 (23.69%)	346 (19.67%)	452 (25.70%)	452 (25.70%)	< 0.001
Statin	2,121 (40.19%)	661 (37.58%)	715 (40.65%)	745 (42.35%)	0.014
Vasoactive	457 (8.66%)	174 (9.89%)	139 (7.90%)	144 (8.19%)	0.076
Vasopressin	23 (0.44%)	12 (0.68%)	5 (0.28%)	6 (0.34%)	0.153
Alb treat	361 (6.84%)	129 (7.33%)	111 (6.31%)	121 (6.88%)	0.484
Antibiotic	620 (11.75%)	250 (14.21%)	209 (11.88%)	161 (9.15%)	< 0.001
Bronchodilator	373 (7.07%)	148 (8.41%)	135 (7.67%)	90 (5.12%)	< 0.001

IQR, interquartile range; SD, standard deviation; BMI, body mass index; SBP, Systolic blood pressure; DBP, diastolic blood pressure; BUN, blood urea nitrogen; CR, creatinine; MCV, mean corpuscular volume; MCH, mean corpuscular hemoglobin; ALT, alanine aminotransferase; AST, aspartate aminotransferase; TG, triglyceride; TC, total cholesterol; HDL, high-density lipoprotein; AF, atrial fibrillation; CHF, congestive heart failure; DM, diabetes mellitus; COPD, chronic obstructive pulmonary disease; CKD, chronic kidney disease; AKI, acute kidney injury; DVT, deep vein thrombosis; ARDS, acute respiratory distress syndrome; ACEI/ARB, angiotensin-converting enzyme inhibitor/angiotensin receptor blocker.

### Relationship between TCBI and in-hospital death in different sex

In the initial unadjusted Model 1, middle and high TCBI were identified as independent risk factors for in-hospital death in ACS patients, with OR of 0.63 (95% CI: 0.49–0.81, *p* < 0.001) and 0.39 (95% CI: 0.30–0.53, *p* < 0.001). In model 2, after adjusting for sex, age, race, higher TCBI was markedly associated with the decreased risk of in-hospital death (Tertile 3 vs Tertile 1: OR (95% CI): 0.61 (0.45, 0.83), *p* = 0.002, *p* for trend = 0.001). In model 3, after adjusting for 17 confounding variables, the TCBI was still independently related to the decreased risk of in-hospital death (Tertile 3 vs Tertile 1: OR (95% CI): 0.67 (0.48, 0.94), *p* = 0.019, *p* for trend = 0.016). Furthermore, when TCBI was considered as a continuous variable in the model for analysis, we observed that for each unit increase in the TCBI, the risk of in-hospital death decreased approximately 38% in Model 1 (*p* < 0.001), 20% in Model 2 (*p* = 0.01), 18% in Model 3 (*p* = 0.026), respectively (Table 2).

**TABLE 2:** Associations of Triglycerides × Total Cholesterol × Body Weight Index (TCBI) with hospital death in acute coronary syndrome (ACS) patients.

	Total population			Male			Female		
	OR (95% CI)	*P*-value	*P* for trend	OR (95% CI)	*P*-value	*P* for trend	OR (95% CI)	*P*-value	*P* for trend
Model 1	–	–	< 0.001	–	–	< 0.001	–	–	0.034
Tertile 1	Reference	–	–	Reference	–	–	Reference	–	–
Tertile 2	0.63 (0.49,0.81)	< 0.001	–	0.60 (0.44, 0.83)	0.002	–	0.66 (0.44, 0.99)	0.047	–
Tertile 3	0.39 (0.30,0.53)	< 0.001	–	0.29 (0.20, 0.43)	< 0.001	–	0.65 (0.42, 1.00)	0.053	–
*Continuous	0.62 (0.52, 0.74)	< 0.001	–	0.48 (0.37, 0.62)	< 0.001	–	0.87 (0.69, 1.09)	0.233	–
Model 2	–	–	0.001	–	–	–	–	–	0.322
Tertile 1	Reference	–	–	Reference	–	0.001	Reference	–	–
Tertile 2	0.78 (0.60, 1.01)	0.057	–	0.78 (0.56, 1.10)	0.154	–	0.76 (0.50, 1.15)	0.193	–
Tertile 3	0.61 (0.45, 0.83)	0.002	–	0.50 (0.33, 0.76)	0.001	–	0.83 (0.52, 1.30)	0.408	–
*Continuous	0.80 (0.67, 0.95)	0.010	–	0.67 (0.52, 0.86)	0.002		1.00 (0.79, 1.25)	0.974	–
Model 3	–	–	0.016	–	–	0.023	–	–	0.373
Tertile 1	Reference	–	–	Reference	–	–	Reference	–	–
Tertile 2	0.82 (0.62, 1.08)	0.162	–	0.85 (0.59, 1.23)	0.381	–	0.78 (0.50, 1.20)	0.257	–
Tertile 3	0.67 (0.48, 0.94)	0.019	–	0.59 (0.38, 0.92)	0.021	–	0.83 (0.51, 1.36)	0.462	–
*Continuous	0.82 (0.69, 0.98)	0.026	–	0.74 (0.57, 0.95)	0.020	–	0.94 (0.74, 1.20)	0.645	–

*Continuous indicated results when Triglycerides × Total Cholesterol × Body Weight Index (TCBI) was analyzed as a continuous variable. Adjust I model adjust for: sex, age, race. Adjust II model adjust for: sex, age, race, heart rate, respiration, CHF, COPD, CKD, DM, stroke, Hemoglobin, CR, BUN, Na, antiplatelet, ACEI/ARB, statin. CHF, Congestive Heart Failure; COPD, Chronic Obstructive Pulmonary Disease; CKD, Chronic Kidney Disease; DM, Diabetes Mellitus; CR, Creatinine; BUN, Blood Urea Nitrogen; ACEI/ARB, Angiotensin-Converting Enzyme Inhibitor / Angiotensin Receptor Blocker.

We further analyzed the association between TCBI tertiles and in-hospital death stratified by sex (Table 2). In males, higher TCBI was significantly associated with a reduced risk of in-hospital death (Tertile 3 vs. Tertile 1 in Model 3: OR 0.59, 95% CI 0.38–0.92, *p* = 0.021, *p* for trend = 0.023). However, this relationship was not significant in females (Tertile 3 vs. Tertile 1 in Model 3: OR 0.83, 95% CI 0.51–1.36, *p* = 0.462, *p* for trend = 0.373). When TCBI was analyzed as a continuous variable, the association remained significant in males (Model 3: OR 0.74, 95% CI 0.57–0.95, *p* = 0.020).

### A Lowess curve analysis of the association between TCBI and in-hospital death

**FIGURE 2:**
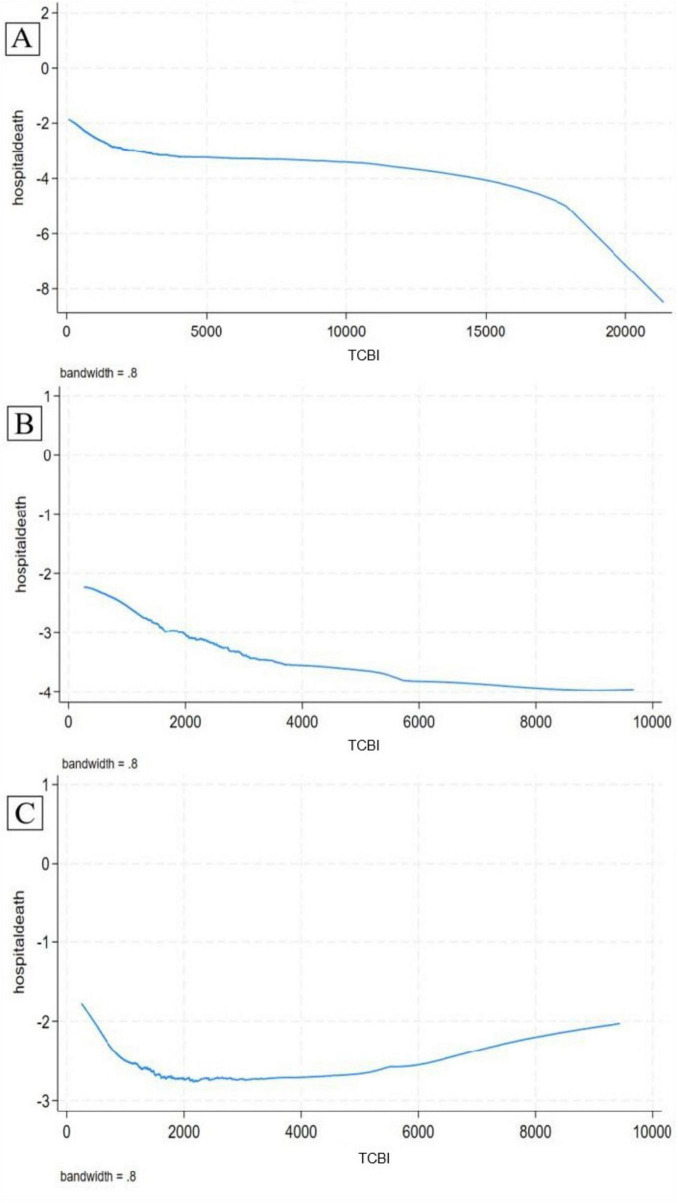
Lowess plots revealed the relationship between Triglycerides × Total Cholesterol × Body Weight Index (TCBI) and hospital death in acute coronary syndrome (ACS) patients **(A)**, male **(B)** and female subgroup **(C)**.

**FIGURE 3:**
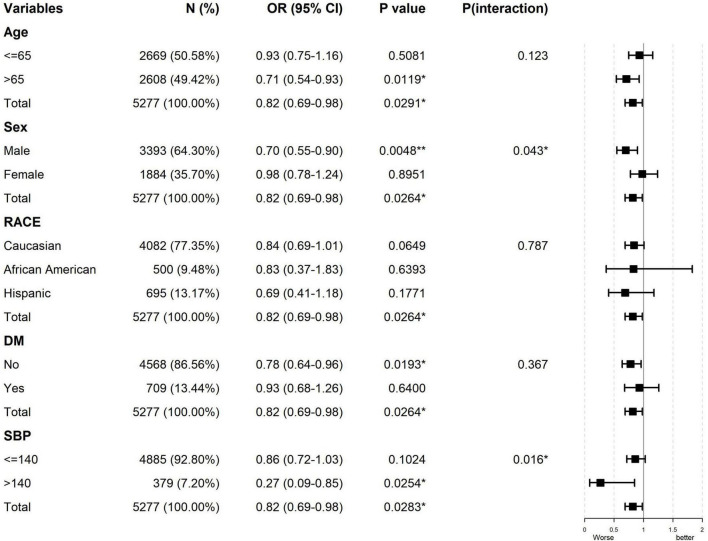
Subgroup analysis for the association of Triglycerides × Total Cholesterol × Body Weight Index (TCBI) and prognosis in acute coronary syndrome (ACS) patients. Adjusted for: sex, age, race, heart rate, respiration, CHF, COPD, CKD, DM, stroke, hemoglobin, CR, BUN, Na, antiplatelet, ACEI/ARB, statin. CHF, congestive heart failure; COPD, chronic obstructive pulmonary disease; CKD, chronic kidney disease; DM, diabetes mellitus; CR, creatinine; BUN, blood urea nitrogen; ACEI/ARB, angiotensin-converting enzyme inhibitor/angiotensin receptor blocker.

The Lowess curve revealed a monotonically decreasing relationship between TCBI and in-hospital death among ACS patients. This trend was particularly pronounced in males but not in females (Figure 2). These findings were consistent with the results of our subgroup analysis, which indicated a significant interaction between sex and in-hospital death (*p* for interaction = 0.043). For males, the odds ratio (OR) was 0.7 (95% CI: 0.55–0.90, *p* = 0.005), suggesting a protective effect of higher TCBI. In contrast, for females, the OR was 0.98 (95% CI: 0.78–1.24, *p* = 0.895), indicating no significant association (Figure 3).

### Subgroup analysis for the association of TCBI and prognosis

The subgroup analysis revealed that, in addition to gender, significant interactions were observed in the SBP subgroups (*p* for interaction = 0.016) (Figure 3). Among patients with SBP below 140 mmHg, higher TCBI was significantly associated with an increased risk of in-hospital death. Conversely, for those with SBP of 140 mmHg or above, no significant correlation was found. Overall, the association between TCBI and clinical outcomes was more pronounced in male patients and those with higher SBP. No significant interactions were observed in the other analyzed subgroups.

## Discussion

This study investigated the relationship between the novel nutritional index TCBI and in-hospital mortality in ACS patients. The main findings were as follows: ① Higher TCBI was independently associated with a decreased risk of in-hospital mortality in ACS patients, especially in male patients. ② The negative correlation between TCBI and in-hospital mortality was significant in the male subgroup but not in the female subgroup. ③ The association between higher TCBI and lower in-hospital mortality was more pronounced in patients with SBP > 140 mmHg compared to those with SBP ≤ 140 mmHg. In conclusion, TCBI was a novel and independent predictor of short-term prognosis in ACS patients, particularly in males and those with SBP > 140 mmHg. These findings suggested that nutritional assessment using TCBI may help risk stratification and personalized management of ACS patients, potentially improving clinical outcomes.

Malnutrition is a common and significant concern among ACS patients, strongly associated with poor clinical outcomes. Recognizing the importance of nutritional assessment, researchers have sought efficient methods to evaluate nutritional status, leading to the emergence of TCBI as a promising tool. TCBI simplifies nutritional assessment by combining total cholesterol, triglycerides, and body weight, normalized by 1,000, providing a more accessible evaluation method in clinical settings. Recent studies have demonstrated TCBI’s potential value in predicting outcomes for ACS patients. Doi et al. found that elevated TCBI was associated with lower mortality rates in 1,987 PCI patients over 7.4 years, including reduced all-cause mortality (HR 0.32, 95% CI 0.19–0.54), cardiovascular mortality (HR 0.29, 95% CI 0.13–0.62), and cancer mortality (HR 0.21, 95% CI 0.07–0.60) (6). Similarly, Maruyama et al. showed that low TCBI predicted higher incidence of major adverse cardiac and cerebrovascular events (HR 2.09, 95% CI 1.36–3.22) in 597 elderly PCI patients over 5 years (10). Importantly, TCBI demonstrated a moderate but significant correlation with the more complex Geriatric Nutritional Risk Index, supporting its validity as a simplified nutritional assessment tool (6). These findings collectively highlight TCBI’s potential as an efficient and valuable predictor of outcomes in ACS patients.

Sex differences in nutrition and metabolism have been widely recognized, such as higher body fat percentage and lower muscle mass in women (11). Moreover, these gender disparities potentially lead to differences in prognosis among coronary heart disease patients (13). However, existing research had not adequately explored the relationship between the TCBI, a nutritional indicator, and the prognosis of patients with ACS, especially within the context of sex stratification.

To address this evidence gap, our study investigated the association between TCBI and short-term prognosis in a large, diverse ACS population, with particular emphasis on potential sex differences. The findings were anticipated to have significant implications for risk stratification and personalized management of ACS patients. Notably, the study revealed that higher TCBI was independently associated with a decreased risk of mortality, with this relationship being particularly pronounced in males and individuals with systolic blood pressure exceeding 140 mmHg. Compared to previous studies that focused on traditional nutritional indicators such as BMI, albumin, or lipid levels alone (14–16), our study introduced a comprehensive index that integrates triglycerides, total cholesterol, and body weight, providing a more holistic assessment of nutritional status and its impact on ACS prognosis. The ease of obtaining these routine clinical parameters and the simplicity of calculating TCBI made it a practical tool for risk stratification and personalized management of ACS patients in various healthcare settings. The subgroup analysis revealed a significant interaction between sex and the predictive value of TCBI, with a stronger association in males compared to females. These findings highlight the importance of considering sex-specific factors when interpreting the prognostic value of nutritional indices in ACS. Furthermore, the more pronounced association between higher TCBI and lower in-hospital mortality in patients with SBP > 140 mmHg suggested that this index may be particularly useful for risk assessment in this high-risk subgroup. Incorporating TCBI into clinical decision-making for hypertensive ACS patients could help identify those who may benefit from more intensive monitoring and targeted interventions. The potential cost-effectiveness of using TCBI as a prognostic tool in ACS management is another advantage, as it relies on readily available clinical data and does not require additional expensive tests. This is particularly relevant for resource-limited settings and healthcare systems aiming to optimize patient outcomes while controlling costs. Future research should focus on validating the predictive value of TCBI in larger, prospective cohorts and exploring its potential as a therapeutic target for improving ACS outcomes.

The negative correlation between TCBI and in-hospital mortality in ACS patients, particularly in the male subgroup, may be attributed to the protective effects of higher lipid levels on cardiovascular function. In a rat model of myocardial infarction, increased triglyceride levels were associated with improved cardiac function and reduced infarct size, possibly through the provision of energy substrates for the ischemic myocardium (17). Furthermore, higher cholesterol levels may contribute to membrane stability and cell survival under stress conditions (18). The sex differences in the predictive value of TCBI for ACS prognosis may be related to the differential effects of sex hormones on lipid metabolism and cardiovascular function. Testosterone has been shown to increase lipoprotein lipase activity and promote triglyceride clearance in animal models (19). Significant sex-related variations in lipid metabolism have also been observed in human clinical studies, where males display greater lipoprotein lipase activity compared to females (20). Recent research (21) has demonstrated that testosterone supplementation improves lipid profiles and reduces inflammatory markers associated with cardiovascular risk. In a mouse model of myocardial infarction, testosterone supplementation reduced infarct size and improved cardiac function (22). These beneficial effects of testosterone on lipid metabolism and cardiovascular protection may explain the stronger association between higher TCBI and lower in-hospital mortality in male ACS patients. In contrast, estrogen has been reported to reduce lipid accumulation and inflammation in animal models of cardiovascular disease (23), with recent studies (24) showing that estrogen’s cardioprotective effects operate through mechanisms independent of lipid parameters, potentially explaining why TCBI’s prognostic value differs between sexes. The more significant correlation between TCBI and reduced in-hospital mortality in patients with blood pressure > 140 mmHg may be due to the potential protective effects of higher lipid levels against hypertension-induced cardiovascular damage. In spontaneously hypertensive rats, a high-fat diet attenuated cardiac hypertrophy and fibrosis by modulating the expression of genes involved in collagen synthesis and degradation (25). Moreover, increased lipid availability might help maintain myocardial energy supply and function under the increased workload imposed by hypertension (17). These findings suggested that higher TCBI may confer greater cardiovascular protection in the presence of hypertension, leading to a more pronounced reduction in in-hospital mortality in this subgroup of ACS patients.

This study has several limitations. First, the retrospective design and potential confounding factors may influence the accuracy of the results. Second, the study population was limited to ICU patients from the eICU-CRD database, which may not be representative of the general ACS population, thus limiting the generalizability of the findings. ACS patients admitted to ICUs typically represent more severe cases with higher acuity and potentially different nutritional profiles compared to those managed in general ward or outpatient settings. This selection bias might affect the applicability of our findings to the broader ACS population. Third, the short-term endpoint of in-hospital mortality was used, and long-term prognostic data were lacking. This focus on in-hospital mortality alone prevents us from evaluating the predictive value of TCBI for long-term outcomes such as major adverse cardiovascular events (MACE), recurrent myocardial infarction, heart failure development, and cardiovascular mortality, which are crucial endpoints in comprehensive ACS prognostic assessment. Additionally, the diagnostic criteria for chronic diseases relied solely on ICD codes from discharge records, which is an inherent limitation of this retrospective study. This approach may lead to potential misclassification and might not fully capture the nuanced clinical presentation of chronic conditions, thus potentially introducing bias in the assessment of comorbidities. The single measurement of TCBI at baseline prevented the assessment of dynamic changes in nutritional status over time. Additionally, the study did not account for the potential impact of treatment strategies, medication adherence and dietary patterns on patient outcomes. Furthermore, the economic constraints of the database limited the ability to conduct repeated measurements of key indicators. Future research should focus on prospective studies with a more comprehensive set of variables, including a broader population, particularly validating our findings in diverse clinical settings including non-ICU ACS patients, and exploring the long-term prognostic value of TCBI in ACS patients. Investigating the potential mechanisms underlying the sex differences in the predictive value of TCBI and its interaction with hypertension could provide valuable insights for personalized risk assessment and management.

Sixth, our study identified chronic diseases based on ICD coding from the eICU database discharge diagnoses. Due to the retrospective nature of the research, there may be uncertainties and potential misclassification in disease diagnosis.

## Conclusion

Our study reveals that TCBI, a novel nutritional index, was independently associated with decreased in-hospital mortality in ACS patients, with a more pronounced effect in males and those with SBP > 140 mmHg. These findings suggested that TCBI could serve as a practical tool for risk stratification and personalized management of ACS patients in clinical settings. Future studies are needed to validate these results, explore the long-term prognostic value of TCBI, and elucidate the underlying mechanisms to refine risk assessment and guide targeted interventions in ACS patients.

## Data Availability

The original contributions presented in the study are included in the article/supplementary material, further inquiries can be directed to the corresponding author.

## Footnotes

1
http://www.R-project.org
